# Occupational Noise Exposure and Incidence of High Fasting Blood Glucose: A 3-Year, Multicenter, Retrospective Study

**DOI:** 10.3390/ijerph18179388

**Published:** 2021-09-06

**Authors:** Seunghan Kim, Byungyoon Yun, Seunghyun Lee, Changyoung Kim, Juho Sim, Ara Cho, Yeonsuh Oh, Jiho Lee, Jinha Yoon

**Affiliations:** 1 Department of Preventive Medicine, Yonsei University College of Medicine, Seoul 03722, Korea; hanpurple12@gmail.com (S.K.); yby3721@yuhs.ac (B.Y.); 2 Office of Research Affairs, Yonsei University, Seoul 03722, Korea; jay1250@yuhs.ac; 3 BigData Center, Ulsan University Hospital, Ulsan 44033, Korea; fingertree@uuh.ulsan.kr; 4 Department of Public Health, Graduate School, Yonsei University, Seoul 03722, Korea; yodasim@yuhs.ac; 5 Department of Occupational Health, Graduate School of Public Health, Yonsei University, Seoul 03722, Korea; aracho88@yuhs.ac; 6 Environmental Health Center, University of Ulsan College of Medicine, Ulsan 44033, Korea; s2lovesky00@naver.com; 7 Department of Occupational & Environmental Medicine, University of Ulsan College of Medicine, Ulsan 44033, Korea; 8 The Institute for Occupational Health, Yonsei University College of Medicine, Seoul 03722, Korea

**Keywords:** occupational noise, fasting blood glucose, Common Data Model, workers’ health examination

## Abstract

The role of hazardous occupational noise exposure on the development of prediabetes is not well researched. We aimed to elucidate exposure to hazardous occupational noise as an independent risk factor for high fasting blood glucose (FBG). Participants exposed/non-exposed to occupational noise were recruited from the Common Data Model cohorts of 2013/2014 from two centers and were followed-up for 3 years. Multivariate time-dependent Cox proportional hazard models were used to estimate hazard ratios (HRs) and 95% confidence intervals (CIs) and were adjusted for various covariates. Pooled HRs were calculated. Among the 43,858 participants of this retrospective cohort study, 37.64% developed high FBG. The mean (standard deviation) age in the cohort was 40.91 (9.71) years. In the fully adjusted models, the HRs of high FBG in the two centers were 1.35 (95% CI: 1.24–1.48) and 1.22 (95% CI: 1.17–1.28), and the pooled HR was 1.28 (95% CI: 1.16–1.41). A Kaplan–Meier plot of high FBG incidence by occupational noise exposure showed significant results (*p* < 0.001). We found that occupational noise exposure is significantly associated with high FBG. Preventing exposure to hazardous noise in the work environment may help reduce the risk for prediabetes among workers.

## 1. Introduction

Prediabetes can cause cardiovascular diseases such as coronary artery disease and heart failure [1]. Patients with glycemic dysregulation may also develop fatty liver, cognitive dysfunction, sleep disorder, sex hormone deficiency, or cancer. Independently, prediabetes can increase the risk of type 2 diabetes mellitus by approximately 3–10 times [2]. Managing diabetes and preventing the progression of prediabetes incurs heavy medical costs. The total medical expenditure on diagnosed diabetes and prediabetes in the United States (US) during 2017 was estimated to be $404 billion, of which $43.4 billion, $327.2 million, and $31.7 billion were spent on prediabetes, diagnosed diabetes, and undiagnosed diabetes, respectively [3]. Furthermore, the total yearly direct expenses of diabetes in Korea were $260 million in 2009, according to the Korea National Health and Nutrition Examination Survey [4].

According to the American Diabetes Association, patients with prediabetes have impaired fasting blood glucose (FBG) levels ranging from 100~125 mg/dL, impaired glucose tolerance with 2-h post prandial blood glucose levels from 140–199 mg/dL, or both [5]. Although some studies show that impaired glucose tolerance is more strongly associated to cardiovascular disease than impaired FBG [6,7,8], both parameters emerged as independent predictors of cardiovascular disease and all-cause mortality in the Australian Diabetes, Obesity, and Lifestyle Study [9]. A meta-analysis also corroborated the association of impaired FBG with cardiovascular disease risk [10]. Furthermore, the prevalence of impaired FBG is increasing worldwide. According to the International Diabetes Federation, global prevalence of impaired FBG was approximately 352.1 million (7.3% of all adults) in 2017 and is estimated to increase to 587 million (8.3% of all adults) in 2045 [11]. In Korea, the number of individuals with impaired FBG was 8.3 million in 2016, comprising 24.8% of all Korean adults aged over 30 years. This number increased to 8.71 million (25.3% of all Korean adults aged over 30 years) in 2018 [12,13].

Impaired FBG has several well-known risk factors including demographic and lifestyle factors. According to the International Diabetes Federation 2018 atlas, in 2017, an estimated 221 million men and 204 million women were diagnosed with type 2 diabetes [14]. A meta-analysis of cohort studies of obesity and the risk of diabetes recorded overall relative risk of 7.19 (95% CI: 5.74–9.00) [15]. Lifestyle factors including physical inactivity, smoking, low alcohol consumption and high sedentary life were associated with the increased risk of type 2 diabetes [16]. On the other hand, since the role of occupational environmental factors on the development of impaired FBG is not clearly established, it is important to examine any such associations because workers are repeatedly exposed to occupational risk factors.

Noise is an occupational environmental risk factor associated with cardiovascular disease, tinnitus, balance disorder, sleep disorder, and obesity [17,18,19]. To minimize harmful effects of noise exposure, the National Institute for Occupational Safety and Health (NIOSH) declared that the recommended exposure limitation (REL) of noise is 85 dBA over 8 h, calculated as a time-weighted average [20]. The American Conference of Governmental Industrial Hygienists also recommended the same REL (85 dBA) [21]. However, approximately 22 million US workers are still being exposed to hazardous occupational noise that exceeds the REL [17], warranting the need to establish the effects of noise on workers’ health.

A previous study that showed the relationship between noise exposure and hyperglycemia reported that exposure to hazardous noise (≥85 dBA) increased the risk for hyperglycemia (relative risk [RR] 1.80; 95% confidence interval [CI]: 1.04, 3.10). That study defined participants with hyperglycemia as those diagnosed with prediabetes, who were undergoing hypoglycemic drug therapy and had an FBG level ≥ 100 mg/dL [22]. However, that study had a small sample size, contained inescapable immortal time bias, and did not adjust for co-exposure factors. More comprehensive study design and analysis with inclusion of larger sample size, which could overcome the previous limitations, were needed to clarify the relationship between occupational noise exposure and increased risk of high FBG levels.

Hence, our current study aimed to elucidate the relationship between occupational noise exposure and the incidence of high FBG levels. Cohort were followed-up for three years, and we calculated the incidence of high FBG between the noise exposure and non-exposure group. We hope this study may overcome previous knowledge gap.

## 2. Materials and Methods

### 2.1. Study Population

In this study, we used health examination data from Common Data Models (CDMs) of two hospitals, namely Severance Hospital and Ulsan University Hospital. A CDM is a method to organize and standardize different data obtained from various institutions. Nine hospitals in Korea currently employ the distributed CDM using Korea workers’ health examination data. We used the baseline health examination data from 2013 or 2014 and the corresponding follow-up data from 2016 or 2017, resulting in a maximum follow-up period of 3 years. Among the 24,370 participants initially recruited from Severance Hospital, those with existing impaired FBG (*n* = 7025) were excluded. We also excluded participants who were lost to follow-up (*n* = 3055) and those whose workplaces were not exposed to occupational noise (*n* = 2358). Among the 60,743 participants recruited from Ulsan University Hospital, those with existing impaired FBG (*n* = 11,936), participants lost to follow-up (*n* = 7023), and those who worked at companies without occupational noise exposure (*n* = 9858) were excluded. Finally, 11,932 and 31,926 participants from Severance Hospital and Ulsan University Hospital, respectively, were enrolled in the study.

### 2.2. Definitions of Covariates

All participants fasted overnight before undertaking the annual health examinations. Venous samples were collected to measure blood glucose levels. High FBG was defined as an FBG ≥ 100 mg/dL and a positive response to the questions, “Have you been diagnosed with diabetes by a physician?” or “Are you taking any drugs for diabetes?”.

Participants who were exposed to occupational noise that exceeded the REL (85 dBA) at any period during the follow-up were classified into the “noise exposure group,” while the rest were classified into the “non-exposure group.” Noise levels were measured on a sound level meter that complied with the American National Standards Institute’s requirements determined by work environment assessment experts. The Korean government announced the Article 125 (Working Environment Monitoring) forcing that government-certificated industrial hygienists should monitor workplace environment including noise exposure [23]. They visit each industry and assess the possibility of noise exposure. The noise exposure workers are registered on the database of Korea Occupational Safety and Health Agency. The noise exposure workers were double-checked by physician of occupational environmental medicine prior to health examination. Data regarding workers’ exposure to noise were collated from the database.

The relevant covariates included age, sex, smoking history, BMI, exercise, alcohol consumption, hypertension, and cardiovascular-related risk factors. Participants who had smoked <5 packs in their entire life were classified as “non-smokers;” the rest were classified as either “ex-smokers” or “current smokers”, according to the current smoking habit. BMI was grouped into four categories, namely “underweight (<18.5)”, “normal (18.5–22.0)”, “overweight (23–24.9)”, and “obese (≥25)” according to the classification provided by the Korean Society for the Study of Obesity [24]. Participants who performed high or medium intensity exercises more than twice a week were classified as the “exercise group,” while the rest were classified as the “non-exercise group.” Alcohol consumption was defined as the consumption of more than seven alcoholic drinks per week for men and more than five alcoholic drinks per week for women.

Participants who answered positively to the questions, “Have you been diagnosed as hypertensive by a physician?” or “Have you been prescribed a drug for hypertension?”, had a systolic blood pressure ≥140 mmHg, or had a diastolic blood pressure ≥90 mmHg were classified as hypertensive. Blood pressure was measured by trained nurses using an automated device. If high blood pressure was observed in the first recording, the blood pressure was measured again after a 10-min rest.

Finally, workers exposed to cardiovascular-related risk factors, such as carbon monoxide, nitric dioxide, cyanide compounds, antimony compounds, carbon disulfide, trichloroethylene, ethylene glycol dinitrate, acetonitrile, methyl chloroform, dichlorofluoromethane, dichloromethane, nitroglycerin, vibration, high or low pressure, and night shift, were classified as the “exposure-positive group.” This was in accordance with the Korean Occupational Safety of Health Act [25]. The number of cardiovascular risk-related exposures was used as a covariate in the Cox proportional hazard models.

### 2.3. Statistical Analysis

The distribution of participants’ characteristics was described using t-tests for nominal variables and chi-square tests for continuous variables. Crude hazard ratios (HRs) with 95% CIs and adjusted HRs with 95% CIs were calculated using multivariate time-dependent Cox proportional hazard models. The data from both hospitals were analyzed using the same statistical methods and were combined by pooling the HRs. The survival ratio of participants with high FBG was drawn using Kaplan–Meier plots. Stratification analysis was performed by stratifying time invariant variables, which are age and sex. Participants were classified into 2 groups with age 40-years-old in stratification analysis. All tests were two-sided, and a *p*-value less than 0.05 was considered statistically significant. All statistical analyses were performed using R program, version 4.0.2 (R Foundation for Statistical Computing, Vienna, Austria). The “survival” statistical package was used for the Cox proportional hazard models, and “metafor” was used for calculating pooled HRs

## 3. Results

Baseline characteristics of participants from the noise exposure and non-exposure groups are presented in Table 1. The overall mean age and standard deviation (SD) was 40.91 years and 9.71 years. The mean ages (SDs) in the noise exposure and non-exposure groups were 43.97 years (SD = 9.81 years) and 38.17 years (SD = 9.62 years), respectively (*p* < 0.001). Men comprised 96.04% and 62.15% of the noise exposure and non-exposure groups, respectively. The percentage of ex-smokers and current smokers was greater in the noise exposure group (27.26%, 45.43%) than in the non-exposed group (17.63%, 22.74%). Moreover, significantly more participants were overweight or obese in the noise exposure group (30.18%, 28.04%) than participants in the non-exposure group (22.88%, 23.55%). Similarly, alcohol was consumed by more participants in the noise exposure group (53.54%) than participants in the non-exposure group (37.48%). Most participants in the noise exposure (89.61%) and non-exposure (91.89%) groups were not hypertensive. However, the percentage of participants who performed physical exercise was greater in the noise exposure group (85.35%) than in the non-exposure group (67.67%). In the noise exposure group, most (60.55%) participants were exposed to cardiovascular risk factors, whereas in the non-exposure group, most participants (86.34%) were not exposed to cardiovascular risk factors. Baseline characteristics of each hospital are summarized in Supplementary Table S1.

**Figure 1:**
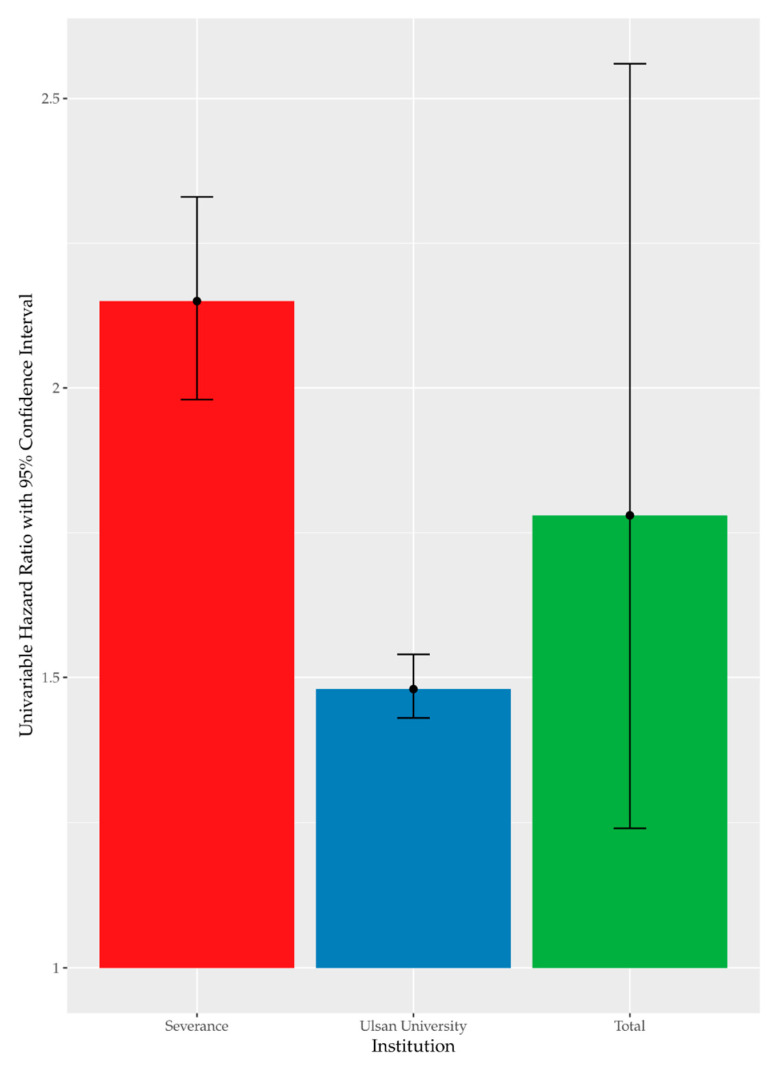
Univariate time-dependent Cox analysis of high FBG by noise exposure.

**Figure 2:**
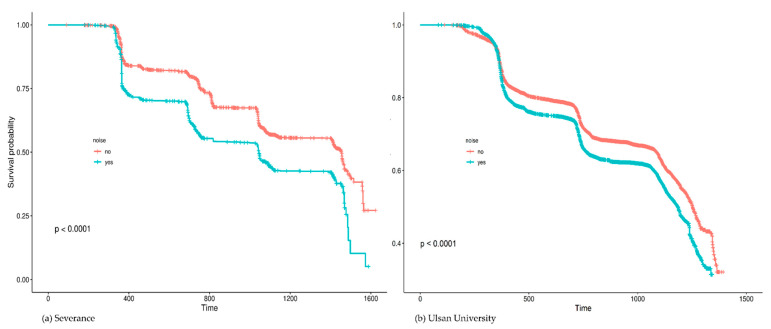
Kaplan–Meier plots of high fasting blood glucose by occupational noise exposure.

High FBG was recorded in 37.64% (*n* = 16,509) of the enrolled participants. Figure 1 summarizes the results of univariate Cox analysis and the crude HRs of high FBG by occupational noise exposure noted in the cohorts from each hospital (Severance Hospital, HR = 2.15 [95% CI:1.98–2.33]; Ulsan University Hospital, HR = 1.48 [95% CI:1.43–1.54]; and overall (pooled HR = 1.78 [95% CI:1.24–2.56]). Figure 2 shows the Kaplan–Meier plot obtained by indicating prediabetes diagnosis at a health examination as an “Event.” In this plot, the period between the baseline and follow-up is termed as “Follow-up Duration,” and data from participants lost to follow-up are termed as “Censored.” The survival rates of the noise exposure and non-exposure groups significantly differed (*p* < 0.0001).

The adjusted models of the multivariate time-dependent Cox proportional hazard analysis are summarized in Table 2. The adjusted HRs of occupational noise exposure were 1.35 (95% CI: 1.24–1.48) in Severance Hospital and 1.22 (95% CI: 1.17–1.28) in Ulsan University Hospital. The pooled HR of high FBG due to occupational noise exposure was 1.28 (95% CI: 1.16–1.41). In addition to noise exposure, older age, male sex, current smoking status, overweight, obesity, alcohol consumption, and hypertension were associated with an increased risk of impaired FBG in the entire cohort. The number of exposures related to cardiovascular risk and past smoking history were not significantly related to an increased risk of impaired FBG levels. Similar trends were observed in each hospital. Supplementary Table S2 summarizes multivariate time-dependent Cox proportional hazard models of each hospital.

In stratification analysis, adjusted HRs of high FBG by occupational noise exposure were statistically significant in male group, old group, and young group, with pooled HR of 1.28 (95% CI: 1.16–1.41), 1.33 (95% CI: 1.17–1.52), and 1.25 (95% CI: 1.17–1.33), respectively. The female group, however, showed no statistical significance (pooled HR 1.03, 95% CI: 0.85–1.25). The results of stratification analysis are shown in Table 3.

## 4. Discussion

This study found a significant relationship between exposure to hazardous occupational noise and the incidence of high FBG. This association was significant in the pooled cohort and in the two cohorts from different centers. Even after adjusting for covariates, the increase in the risk for high FBG because of occupational noise exposure remained statistically significant. Similar results were obtained after omitting bias. In stratification analysis, all groups except the female group showed a significant relationship between exposure to hazardous occupational noise and the increased risk of high FBG. The insignificant results of the female group may have been due to the small sample size of females.

Since prediabetes is closely associated with diabetes and leads to various complications, preventing the progression of hyperglycemia during preliminary stages is important [2,10]. Furthermore, there is a need to identify how working conditions impact the risks for cardiovascular diseases including diabetes to develop efficient preventive measures. This knowledge could also help in raising awareness of cardiovascular diseases and diabetes, aiding in the early diagnosis of these conditions, and development of preventive strategies. Workers, particularly those in aging populations or those exposed to occupational risk factors, may be particularly susceptible to cardiovascular diseases and diabetes [26]. Our study shows a significant association between occupational noise exposure and the risk for prediabetes. Therefore, it is critical to identify ways to improve hyperglycemia by controlling noise exposure and detect prediabetes early through screening of vulnerable working groups.

However, studies about the benefit of personal protective equipment (PPE) or Hearing Loss Prevention Programs on occupational noise exposure have been mostly focused on hearing loss as the outcome [27,28]. There is a lack of intervention studies that clarify the association between noise intervention and improvement of HBG. On the other hand, there is abundant literature emphasizing the effect of lifestyle interventions on diabetes control and prevention. To control expenses and enhance population health, the Diabetes Prevention Program (DPP) for workplace prevention of type 2 diabetes mellitus was advocated. In 2002, the DPP Research Group found that a 7% weight loss and 150 min of physical exercise per week reduced the 3-year incidence of type 2 diabetes mellitus in persons with prediabetes by 58% [29]. Therefore, intervention studies that explore the impact of controlling occupational noise exposure by using PPE to reduce hyperglycemia should be conducted in the future.

There are several possible explanations for our findings, emotional stress being the most well-known mechanism. Emotional stress derived from noise exposure causes hyperglycemia in two ways. The first is a hormonal mechanism. Normally, blood glucose levels are controlled by the Hypothalmus-Pituitary-Adrenal axis that regulates the hormone levels of the body. However, if insulin resistance of the cells increases, insulin is unable to efficiently lower the blood glucose levels, leading to hyperglycemia. When albino rats were exposed to noise, the plasma level of their stress hormones, such as corticosterone, adrenaline, and noradrenaline, increased [30]. If emotional stress triggers counter-regulatory hormones, such as cortisol and adrenaline, it may increase insulin resistance and cause the blood glucose to be underutilized and produced excessively [31]. Second, stress can lead to unhealthy lifestyle habits such as smoking and alcohol consumption [32]. Unhealthy lifestyles often cause obesity and high BMI. Since the acquired resistance to stimulate glucose transportation in skeletal muscles is associated with obesity, an unhealthy lifestyle eventually promotes the development of type 2 diabetes mellitus. Additionally, excessive fatty acids disrupt the insulin signaling pathway, leading to insulin resistance [33]. Furthermore, growth hormone-mediated adipose tissue lipolysis promotes hepatic insulin resistance that may cause impaired fasting glucose levels [34].

This study has several limitations. First, although all participants were followed-up every year, it was not possible to know the exact time of incidence of hyperglycemia owing to the limitation of the examination data. Lack of this knowledge may have overestimated the association because prediabetes is asymptomatic. Second, non-occupational noise, such as noise from a participant’s living area was not taken into account. Third, quantitative correlations were not considered. Further study is necessary to investigate the dose–response that may vary based on the duration of exposure and loudness of the noise. Lastly, health examination data are susceptible to bias caused by the healthy worker effect. However, this study’s outcome was high FBG, a non-severe condition that may not cause workers to retire. Further studies that include retired workers should be conducted to minimize this bias.

Despite its limitations, this study has several strengths. First, we used a large cohort and data from two centers. Second, all relevant covariates including age, sex, smoking history, BMI, exercise, alcohol consumption, and hypertension were adjusted in the analyses. Moreover, the number of exposures related to cardiovascular risk, which was not utilized in previous studies, was adjusted. Third, using CDM, this study analyzed a wide range of occupations. This allowed our study the advantage of standardizing the data of participants using the same analytic approach, making it technically superior. Fourth, workplaces without hazardous noise exposure were excluded from our cohort. We were able to compare and assess workers in similar situations. After narrowing down the workplaces with noise exposure, we classified the noise exposed and non-exposed groups from companies with noise exposure and compared them to reduce selection bias. Finally, we minimized the effect of immortal time bias. Time-dependent Cox proportional models were used to reduce this bias by splitting the period of exposure and non-exposure among participants [35].

## 5. Conclusions

This study found a meaningful relationship between exposure to hazardous occupational noise and high FBG levels. Since excessive noise is preventable, it is possible to lower diabetes risk by managing noise exposure. Our report can inform the evidence used to develop such recommendations. Further intervention studies should explore the benefit of occupational noise control on cardiovascular disease risk.

## Figures and Tables

**Table 1:** Demographic characteristics of Study Population.

	Unexposed Group	Noise Exposed Group	*p*-Value
Age, Mean (SD)			<0.001
	38.17(9.62)	43.97(9.81)	
Sex			<0.001
Male	14,390 (62.15%)	19,883 (96.04%)	
Female	8765 (37.85%)	820 (3.96%)	
Smoking history			<0.001
Non-smoker	13,806 (59.62%)	5654 (27.31%)	
Ex-smoker	4083 (17.63%)	5643 (27.26%)	
Current smoker	5266 (22.74%)	9406 (45.43%)	
BMI			<0.001
Underweight	1474 (06.37%)	226 (01.09%)	
Normal	10,930 (47.20%)	8423 (40.68%)	
Overweight	5298 (22.88%)	6249 (30.18%)	
Obese	5453 (23.55%)	5805 (28.04%)	
Alcohol consumption			<0.001
Yes	8678 (37.48%)	11,085 (53.54%)	
No	14,477 (62.52%)	9618 (46.46%)	
Hypertension			<0.001
Yes	1878 (08.11%)	2151 (10.39%)	
No	21,277 (91.89%)	18,552 (89.61%)	
Physical exercise			<0.001
Yes	15,668 (67.67%)	17,670 (85.35%)	
No	7487 (32.33%)	3033 (14.65%)	
Cardiovascular related exposure			<0.001
Yes	3162 (13.66%)	12,536 (60.55%)	
No	19,993 (86.34%)	8167 (39.45%)	

**Table 2:** 95% Confidence Interval Hazard Ratio from multivariate time-dependent Cox analysis.

Variables	Severance	Ulsan University	Total
Hazardous noise exposure			
	1.35 (1.24–1.48)	1.22 (1.17–1.28)	1.28 (1.16–1.41)
Age			
	1.02 (1.02–1.02)	1.02 (1.02–1.03)	1.02 (1.02–1.03)
Sex			
Male	1.00 (Reference)	1.00 (Reference)	1.00 (Reference)
Female	0.70 (0.64–0.76)	0.53 (0.48–0.58)	0.61 (0.46–0.80)
Smoking history			
Non-smoker	1.00 (Reference)	1.00 (Reference)	1.00 (Reference)
Ex-smoker	1.10 (1.00–1.22)	1.01 (0.96–1.06)	1.04 (0.96–1.13)
Current smoker	1.29 (1.18–1.40)	1.14 (1.08–1.19)	1.20 (1.07–1.36)
BMI			
Underweight	0.71 (0.62–0.82)	0.80 (0.66–0.97)	0.74 (0.66–0.83)
Normal	1.00 (Reference)	1.00 (Reference)	1.00 (Reference)
Overweight	1.18 (1.09–1.28)	1.13 (1.08–1.18)	1.14 (1.10–1.19)
Obese	1.44 (1.33–1.56)	1.29 (1.24–1.35)	1.36 (1.22–1.51)
Alcohol consumption			
Yes	1.35 (1.26–1.44)	1.20 (1.16–1.25)	1.27 (1.13–1.42)
No	1.00 (Reference)	1.00 (Reference)	1.00 (Reference)
Hypertension			
Yes	1.14 (1.03–1.25)	1.36 (1.29–1.44)	1.26 (1.10–1.49)
No	1.00 (Reference)	1.00 (Reference)	1.00 (Reference)
Physical exercise			
Yes	1.00 (Reference)	1.00 (Reference)	1.00 (Reference)
No	0.96 (0.90–1.01)	0.95 (0.90–1.00)	0.95 (0.92–0.99)
The number of exposures related to cardiovascular risk			
	0.94 (0.90–0.99)	1.01 (0.99–1.02)	0.98 (0.92–1.04)

**Table 3:** 95% Confidence interval hazard ratio of high FBG from stratification analysis.

Variables		Severance	Ulsan University	Total
Sex	Male	1.36 (1.24–1.49)	1.23 (1.17–1.28)	1.28 (1.16–1.41)
	Female	1.10 (0.84–1.44)	0.97 (0.74–1.27)	1.03 (0.85–1.25)
Age	≥40 (old)	1.44 (1.29–1.61)	1.26 (1.20–1.33)	1.33 (1.17–1.52)
	<40 (young)	1.27 (1.11–1.46)	1.24 (1.14–1.34)	1.25 (1.17–1.33)

## Data Availability

Data sharing not applicable.

## Supplementary Materials

The following are available online at https://www.mdpi.com/article/10.3390/ijerph18179388/s1, Table S1. Demographic Characteristics of Study Population in each hospital; Table S2. Multivariate time dependent Cox proportional hazard models of each hospital.

## Abbreviations

FBG	fasting blood glucose
CDM	Common Data Model
REL	recommended exposure limitation
DPP	Diabetes Prevention Program
PPE	personal protective equipment
BMI	body mass index
HR	hazard ratio
CI	confidence interval
RR	relative risk
