# Supplementary material for: Occupational Noise Exposure and Incidence of High Fasting Blood Glucose: A 3-Year, Multicenter, Retrospective Study
Source: Int J Environ Res Public Health. 2021 Sep 6;18(17):9388. doi: 10.3390/ijerph18179388 (PMC8431404; doi:10.3390/ijerph18179388)
Supplement: Supplementary file 1 [file ijerph-18-09388-s001.zip › ijerph-1334231-supplementary.pdf]

International Journal of
Environmental Research and Public Health

MDPI

Supplmentary:

Table S1. Demographic Characteristics of Study Population in each hospital.

	Severance (n=11,932)			Ulsan University (n=31,962)		
	Unexposed group	Noise exposed group	p-value	Unexposed group	Noise exposed group	p-value
Age, Mean (SD)			<0.001			<0.001
	35.97 (9.30)	40.28 (10.91)		39.84 (9.84)	44.24 (10.26)	
Sex			<0.001			<0.001
Male	3858 (37.86%)	1555 (89.32%)		10532 (81.24%)	18328 (96.66%)	
Female	6333 (62.14%)	186 (10.68%)		2432 (18.76%)	634 (3.34%)	
Smoking history			<0.001			<0.001
Non-smoker	7573 (74.31%)	731 (41.99%)		6233 (48.08%)	4923 (25.96%)	
Ex-smoker	894 (8.77%)	331 (19.01%)		3189 (24.60%)	5312 (28.02%)	
Current-smoker	1724 (16.92%)	679 (39.00%)		3542 (27.32%)	8727 (46.02%)	
BMI			<0.001			<0.001
underweight	1121 (11.00%)	38 (2.18%)		353 (2.72%)	188 (0.99%)	
normal	5780 (56.72%)	767 (44.06%)		5150 (39.73%)	7656 (40.38%)	
overweight	1729 (16.97%)	438 (25.16%)		3569 (27.53%)	5811 (30.65%)	
obese	1561 (15.31%)	498 (28.60%)		3892 (30.02%)	5307 (27.98%)	
Alcohol consumption			<0.001			<0.001
Yes	2473 (24.27%)	828 (47.56%)		6205 (47.86%)	10257 (54.09%)	
No	7718 (75.73%)	913 (52.44%)		6759 (52.14%)	8705 (45.91%)	
Hypertension			<0.001			<0.001
Yes	742 (7.28%)	274 (15.74%)		1136 (8.76%)	1877 (9.90%)	
No	9449 (92.72%)	1467 (84.26%)		11828 (91.24%)	17085 (90.10%)	
Physical exercise			<0.001			0.625
Yes	4303 (42.22%)	1011 (58.07%)		11365 (87.67%)	16659 (87.85%)	
No	5888 (57.78%)	730 (41.93%)		1599 (12.33%)	2303 (12.15%)	
Cardiovascular related exposure			0.028			<0.001

Yes	1835 (18.01%)	275 (15.80%)	1327 (10.24%)	12261 (64.66%)
No	8356 (81.99%)	1466 (84.20%)	11637 (89.76%)	6701 (35.34%)

Table S2. Multivariate time dependent Cox proportional hazard models of each hospital.

	Severance				Ulsan University			
**Variables**	**Crude Model**	**Model 1**	**Model 2**	**Final Model**	**Crude Model**	**Model 1**	**Model 2**	**Final Model**
Hazardous noise exposure	2.15 (1.98-2.33)	1.38 (1.27-1.50)	1.32 (1.22-1.44)	1.35 (1.24-1.48)	1.48 (1.43-1.54)	1.22 (1.18-1.27)	1.22 (1.18-1.27)	1.22 (1.17-1.28)
Age		1.02 (1.02-1.02)	1.02 (1.02-1.02)	1.02 (1.02-1.02)		1.02 (1.02-1.03)	1.02 (1.02-1.03)	1.02 (1.02-1.03)
Sex								
Male		1.00 (Reference)	1.00 (Reference)	1.00 (Reference)		1.00 (Reference)	1.00 (Reference)	1.00 (Reference)
Female		0.49 (0.46-0.52)	0.68 (0.63-0.74)	0.70 (0.64-0.76)		0.42 (0.38-0.46)	0.53 (0.49-0.59)	0.53 (0.48-0.58)
Smoking history								
Non-smoker			1.00 (Reference)	1.00 (Reference)			1.00 (Reference)	1.00 (Reference)
Ex-smoker			1.10 (1.00-1.22)	1.10 (1.00-1.22)			1.06 (0.96-1.06)	1.01 (0.96-1.06)
Current-smoker			1.28 (1.18-1.40)	1.29 (1.18-1.40)			1.13 (1.08-1.19)	1.14 (1.08-1.19)
BMI								
underweight			0.71 (0.62-0.82)	0.71 (0.62-0.82)			0.81 (0.66-0.98)	0.80 (0.66-0.97)
normal			1.00 (Reference)	1.00 (Reference)			1.00 (Reference)	1.00 (Reference)
overweight			1.19 (1.10-1.28)	1.18 (1.09-1.28)			1.14 (1.09-1.19)	1.13 (1.08-1.18)
obese			1.47 (1.36-1.58)	1.44 (1.33-1.56)			1.33 (1.27-1.39)	1.29 (1.24-1.35)

Alcohol consumption				
Yes	1.35 (1.26-1.45)	1.35 (1.26-1.44)	1.22 (1.18-1.27)	1.20 (1.16-1.25)
No	1.00 (Reference)	1.00 (Reference)	1.00 (Reference)	1.00 (Reference)
Hypertension				
Yes		1.14 (1.03-1.25)		1.36 (1.29-1.44)
No		1.00 (Reference)		1.00 (Reference)
Physical exercise				
Yes		1.00 (Reference)		1.00 (Reference)
No		0.96 (0.90-1.01)		0.95 (0.90-1.00)
The number of exposures related to cardiovascular risk		0.94 (0.90-0.99)		1.01 (0.99-1.02)
